# Decoding the genetic blueprints of neurological disorders: disease mechanisms and breakthrough gene therapies

**DOI:** 10.3389/fneur.2025.1422707

**Published:** 2025-04-11

**Authors:** Umar Saeed, Zahra Zahid Piracha, Muhammad Nouman Tariq, Syed Shayan Gilani, Maria Rauf, Laiba Razaq, Muhammad Kaleem Iftikhar, Amna Maqsood, Syed Muhammad Ahsan

**Affiliations:** 1 Operational Research Center in Healthcare, Near East University, Nicosia, Türkiye; 2 Foundation University School of Health Sciences (FUSH), Foundation University Islamabad, Islamabad, Pakistan; 3 International Center of Medical Sciences Research (ICMSR), Islamabad, Pakistan; 4 International Center of Medical Sciences Research (ICMSR), Austin, TX, United States; 5 International Center of Medical Sciences Research (ICMSR), Essex, United Kingdom; 6 Akhtar Saeed Medical & Dental College, Lahore, Pakistan; 7 University College of Medicine and Dentistry, Lahore, Pakistan; 8 Punjab Institute of Neurosciences, Lahore, Pakistan; 9 Dow University of Health Sciences, Karachi, Pakistan

**Keywords:** ataxias, autosomal recessive ataxia, mitochondrial cerebellar ataxia, multiple system atrophy, idiopathic late-onset cerebellar ataxia, hereditary spastic paraplegias, Alzheimer’s disease, vascular dementia

## Abstract

Neurological disorders pose a rapidly growing global health burden, significantly affecting cognitive and motor functions with profound societal repercussions. This comprehensive review probe into the genetic foundations of various neurological conditions while exploring innovative RNA-based therapeutics particularly gene therapies as cutting edge treatment strategies. Through an in-depth analysis of existing literature, the study examines the genetic landscape, disease mechanisms, and gene-based intervention possibilities across a range of neurological disorders, including Cerebellar Ataxias, Autosomal Recessive Ataxia, Mitochondrial Cerebellar Ataxia, Multiple System Atrophy (MSA), Idiopathic Late-Onset Cerebellar Ataxia, Hereditary Spastic Paraplegias, Alzheimer’s Disease, Vascular Dementia, Lewy Body Dementia, Frontotemporal Dementias, Inherited Prion Diseases, and Huntington’s Disease. It uncovers the intricate network of genetic mutations driving these disorders, shedding light on their mechanisms and uncovering promising therapeutic targets. The review also highlights the remarkable potential of RNA-based therapeutics, with gene therapies standing at the forefront of precision treatment approaches. By offering an up-to-date understanding of the genetic intricacies and emerging therapeutic possibilities in neurological disorders, this study significantly contributes to the advancement of precision medicine in neurology. It also paves the way for future research and clinical applications aimed at improving patient care and outcomes.

## Introduction

Neurological conditions pose a significant and escalating global health concern, causing substantial impairment to cognitive and motor functions (1). Stroke stands as the second primary contributor to global mortality and the third significant factor in premature mortality and disability, measured in DALYs (Disability-Adjusted Life Years).

Cerebrovascular disease holds the position of being the largest contributor among neurologic conditions, accounting for 4.1% of the total global DALY. Following closely are headaches at 0.97% (migraine 0.90%, tension-type headache 0.07%), other neurologic disorders at 0.72%, epilepsy at 0.70%, Alzheimer’s disease (AD), and other dementias at 0.46%, Parkinson’s disease (PD) at 0.08%, and multiple sclerosis (MS) at 0.04%. Neurological conditions that fall outside the neurologic disorders category encompass meningitis (1.2%), encephalitis (0.29%), and traumatic brain injury (TBI) (2). Neurological disorders, affecting about 15% of the global population, reign as the primary cause of both physical and cognitive disabilities worldwide, with the added concern of the burden from chronic neurodegenerative conditions projected to double in the coming two decades (3). The most significant impact of neurological diseases is observed in countries with low-income and middle-income levels (LMICs) (4).

While substantial advancements have been achieved in addressing genetic diseases, the potential for the development of RNA-based therapeutics to treat conditions like brain tumors, neurodegenerative diseases, stroke, and behavioral disorders is promising. This is particularly notable as these conditions are increasingly acknowledged as disorders linked to RNA metabolism (5). Genes can be classified into two categories: causative genes and associated genes. Causative genes are directly linked to the expected phenotype, typically determined through model systems with a phenotype resembling that in humans. In contrast, associated genes are those where the relationship between the gene and its phenotype cannot be solely attributed to genetic factors (4, 5). Mutations are suggested to be inherent variations contributing to diversity within a species (such as the human genome) and distinctions between different species, with considerations for their advantageous, neutral, or detrimental effects within human genomes and/or across species (6).

Numerous severe neurological conditions resist conventional treatments, necessitating exploration beyond symptomatic relief. Current therapies involve the direct delivery of therapeutic molecules or corresponding genes to the brain. While traditional methods require frequent administration, gene therapy holds promise for longer-term effects from a single infusion. The success of gene therapy hinges on factors such as temporal and spatial precision, dictating whether gene activation is continuous or controlled and whether it is restricted to specific brain areas or cell types. Targeted delivery through focused neurosurgery avoids the blood–brain barrier, enabling precise therapy directed at specific anatomical regions (7).

RNA therapeutics, spanning a spectrum of oligonucleotide-based drugs, such as antisense oligonucleotides (ASOs), small interfering RNAs (siRNAs), short hairpin RNAs (shRNAs), aptamers, microRNA inhibitors (antimiRs), and microRNA mimics (8). This paper concentrates on the clinical and genetic features associated with rare hereditary neurodegenerative conditions, encompassing ataxias, multiple system atrophy, spastic paraplegias, Parkinson’s disease, dementias, motor neuron diseases, and infrequent metabolic disorders.

This primary objective of this article aims to investigate the genetic origins of neurological disorders by identifying relevant mutations. Understanding these genetic factors can shed light on disease mechanisms and inform potential gene-based therapies, fostering advancements in neurology and better patient care. The other objective of this article is to investigate the genetic foundations of neurological conditions, with a focus on identifying genetic mutations linked to neurological diseases. Additionally, the article aims to explore the potential of gene therapeutics in addressing these conditions.

## Cerebellar ataxias

Cerebellar ataxias constitute a spectrum of disorders characterized by cerebellum degeneration and diverse clinical manifestations. These conditions are broadly classified into sporadic and hereditary types. Sporadic ataxias can arise from various causes, including exposure to toxins or structural abnormalities, and may also be of idiopathic origin. In contrast, hereditary ataxias, accounting for 60–75% of cases, exhibit distinct inheritance patterns, necessitating thorough diagnostic assessment (9).

Among hereditary ataxias, Autosomal Dominant Cerebellar Ataxias (ADCAs) represent a subgroup characterized by neurodegenerative processes leading to cerebellar degeneration and disruptions in neural connections. The estimated prevalence of Spinocerebellar Ataxias (SCAs), a subtype of ADCAs, is approximately 1–5 per 100,000 individuals (10). Current data suggests that the pathology of SCAs involves the alteration of native protein functions and deleterious effects stemming from elongated polyglutamine (polyQ) stretches. These disruptions intricately interfere with common cellular processes, contributing to the overall disease progression (10).

Key disruptions encompass transcriptional irregularities, RNA toxicity, toxicity induced by peptides resulting from repeat-associated non-ATG (RAN) translation, dysregulation of the ubiquitin-proteasome system, and impairment of autophagy. Lithium, known for its diverse effects, has been studied for its impact on autophagy, a crucial cellular process in neurodegenerative research. Notably, lithium has shown promise in enhancing autophagy, a potential avenue for mitigating neurodegenerative effects (11).

Temsirolimus, an inhibitor of the mammalian target of rapamycin (mTOR), offers another avenue for modulating autophagy and alleviating toxicity. In the context of Huntington’s disease (HD), mouse models have demonstrated the potential of temsirolimus to enhance autophagy and mitigate associated toxicity. Additionally, caloric restriction, a strategy known to strongly induce autophagy, is being explored as a potential approach to decelerate the progression of neurodegenerative diseases. This involves the pivotal autophagy initiator protein Beclin-1 (12). These insights into the molecular mechanisms underlying cerebellar ataxias and the potential therapeutic interventions provide valuable perspectives for further understanding and addressing these complex neurological conditions. The genetic mutations (Table 1) and consequent classification of cerebellar ataxias are shown (Table 2).

**Table 1:** Genes and therapeutic targets for neurological conditions.

Disorder	Genes identified	Therapeutic targets
Sporadic Ataxias	–	Environmental/Unknown factors
Hereditary Ataxias (SCA)	ATXN1, ATXN2, ATXN3	Motor dysfunction, balance loss; Targeting gene mutations for symptom management
Autosomal Recessive Ataxias (ARA)	FXN (Friedreich’s Ataxia)	Frataxin gene therapy; targeting mitochondrial function and iron–sulfur cluster formation
Mitochondrial Ataxias	POLG, MT-TL1	Targeting mitochondrial dysfunction and protein synthesis
Ataxia Telangiectasia (A-T)	ATM, Mre11-Rad50-Nbs1 (MRN) Complex	DNA repair enhancement, NAD+ levels modulation, mitophagy targeting
Fragile X Syndrome (FXS)	FMR1	CGG trinucleotide repeat expansion treatment, gene silencing
Multiple System Atrophy (MSA)	SNCA, LRRK2, GBA, COQ2, MAPT	α-synuclein aggregation inhibition, LRRK2 mutation targeting, tau protein regulation
Hereditary Spastic Paraplegias (HSP)	–	Gene therapy for axon degeneration, targeting motor function restoration
Parkinson’s Disease (PD)	LRRK2, PARK7, PINK1, SNCA	Rasagiline for neuroprotection, genetic mutation-specific therapies, α-synuclein targeting
Alzheimer’s Disease (AD)	APP, PSEN1, PSEN2, CR1, APOE, CLU	Targeting amyloid plaques, tau protein accumulation, and neuroinflammation
Vascular Dementia (VaD)	APOE, Notch 3, COL4A1, COL4A2	Gene editing for vascular integrity, neuroprotection, targeting vascular risk factors
Frontotemporal Dementia (FTD)	MAPT, GRN, C9orf72, TBK1	Tau protein, progranulin mutation treatment, reducing C9orf72 poly(GA) aggregation
Prion Diseases	PRNP	Targeting prion protein aggregation and replication inhibition
Amyotrophic Lateral Sclerosis (ALS)	SOD1, C9orf72, FUS, TDP-43	Gene silencing for C9orf72 repeat expansions, targeting neuroinflammation and protein aggregation
Huntington’s Disease (HD)	HTT (Huntingtin gene)	Silencing huntingtin, gene editing, reducing polyglutamine toxicity
Multiple Sclerosis (MS)	IL7R, TNF, CD4, CD8, APOE	Immune modulation, cytokine inhibition, targeting demyelination and neuroinflammation
Schizophrenia	DISC1, COMT, NRG1, BDNF, AKT1	Dopamine receptor modulation, neurodevelopmental and synaptic plasticity targeting
Bipolar Disorder	BDNF, GSK3B, CLOCK	Targeting mood stabilization, lithium and GSK3B modulation
Epilepsy	SCN1A, KCNQ2, PCDH19, GABRG2	Ion channel modulation, enhancing GABAergic inhibition, gene therapy for channelopathies
Migraine	CGRP, TRPV1, NOS, MTHFR	CGRP antagonism, ion channel inhibitors, genetic mutations targeting neurotransmitter regulation
Spinal Muscular Atrophy (SMA)	SMN1, SMN2	SMN1 gene replacement therapy, exon 7 skipping for SMN2 activation
Retinitis Pigmentosa (RP)	RHO, RPGR, USH2A, PDE6B	Gene therapy to replace defective rhodopsin, retinal cell regeneration
Charcot–Marie–Tooth Disease (CMT)	PMP22, MPZ, LITAF, GJB1	Gene therapy targeting myelin production, ion channel modulation, cell regeneration
Leber’s Hereditary Optic Neuropathy (LHON)	MT-ND1, MT-ND4, MT-ND6, MT-CO1	Mitochondrial gene therapy to restore optic nerve function
Cerebral Palsy	–	Developmental plasticity, targeting neural circuit repair and neurogenesis
Neurofibromatosis Type 1 (NF1)	NF1 (Neurofibromin)	Targeting Ras/MAPK pathway, inhibitors of MEK/ERK signaling
Tuberous Sclerosis Complex (TSC)	TSC1, TSC2	Targeting mTORC1 inhibition, rapamycin-based therapies for tumor growth regulation
Wilson’s Disease	ATP7B	Gene therapy for copper transport, chaperone proteins for ATP7B folding

**Table 2:** Classification of cerebellar ataxias and key genetic mutations.

Type	Classification	Gene(s) involved	Inheritance pattern	Key symptoms
Sporadic Ataxias	Environmental/Unknown	–	Non-hereditary	Progressive loss of coordination
Hereditary Ataxias (SCA)	Autosomal Dominant	ATXN1, ATXN2, ATXN3	Dominant	Motor dysfunction, balance loss
Autosomal Recessive Ataxias	Autosomal Recessive	FXN (Friedreich’s Ataxia)	Recessive	Gait disturbances, scoliosis
Mitochondrial Ataxias	Mitochondrial	POLG, MT-TL1	Maternal	Cognitive decline, seizures

## Autosomal recessive ataxia

Autosomal Recessive Ataxias (ARAs) represent a group of neurological disorders characterized by cerebellar ataxia resulting from autosomal recessive inheritance patterns. In contrast to Autosomal Dominant Cerebellar Ataxias (ADCAs), where a single mutated gene copy can lead to the manifestation of the disease, ARAs requires both copies of the relevant gene to carry mutations for the condition to manifest (13). The spectrum of ARAs is diverse, encompassing various genetic subtypes with distinct clinical presentations. Unlike sporadic ataxias, which may be influenced by environmental factors, ARAs has a clear genetic basis. These disorders often manifest in childhood or adolescence and progress over time, affecting coordination, balance, and motor skills (13).

One notable example within the realm of ARAs is Friedreich’s ataxia (FRDA), an autosomal recessive ataxia linked to GAA triplet repeat expansion in the frataxin (FXN) gene. Disease severity and onset age correlate with the length of the repeat expansion. Frataxin facilitates iron–sulfur cluster (ISC) formation by enhancing sulfur transfer on the ISC assembly protein ISCU2, acting as an allosteric modulator, highlights its significance in disease pathology (14). Research into therapeutic interventions, such as Omaveloxolone activating Nrf2, enhances mitochondrial function, rebalances redox levels, and alleviates inflammation in Friedreich’s ataxia (FA) models (15).

Ataxia telangiectasia (A-T) is another rare autosomal recessive disorder characterized by uncoordinated movement, telangiectasia, sensitivity to radiation, and cerebellar atrophy. Understanding the molecular mechanisms, including the role of the Mre11-Rad50-Nbs1 (MRN) complex and ATM activation in response to DNA damage, sheds light on potential therapeutic strategies. Addressing DNA repair defects and maintaining proper NAD+ levels are critical in influencing mitophagy and DNA repair enhancement. Certain uncommon disorders, like Niemann-Pick, a lipid storage ailment linked to dementia or psychiatric symptoms, and GM1 gangliosidosis, which can involve dystonia, may exhibit adult onset and follow an autosomal recessive pattern (16).

Fragile X syndrome (FXS) stems from an FMR1 full mutation or loss-of-function variant, typically causing developmental delay, intellectual disability, and behavioral challenges in affected males. A significant percentage (50–70%) of individuals with FXS also have autism spectrum disorder. Diagnosis involves specialized molecular genetic testing, focusing on detecting CGG trinucleotide repeat expansion in FMR1’s 5’ UTR, particularly with abnormal gene methylation for most alleles having over 200 repeats (17).

## Mitochondrial cerebellar ataxia

Mitochondrial Cerebellar Ataxia (MCA) is a neurological condition characterized by disturbances in motor coordination and movement attributed to dysfunctional mitochondria, the cellular organelles responsible for energy production. The condition is marked by a complex interplay between mitochondrial anomalies and cerebellar dysfunction, resulting in impaired coordination and motor skills (18).

Unlike certain types of ataxias, MCA is intricately linked to mitochondrial dysfunction, where disruptions in mitochondrial DNA (mtDNA) contribute to the compromised energy metabolism within cells (19). It can arise from mutations in mitochondrial DNA (mtDNA) or defects in nuclear DNA (nDNA) genes functionally relevant for oxidative phosphorylation (OXPHOS). These disruptions manifest as a communication breakdown between the cerebellum, the brain region responsible for coordinating movements, and the affected mitochondria. The resultant ataxic symptoms include challenges in walking, balancing, and fine motor control, akin to a choreographed routine where the performers lose synchronization (20).

The landscape of therapeutic options for mitochondrial diseases is currently characterized by limited effectiveness. Individuals diagnosed with primary mitochondrial disorders, however, may find potential benefits from specific supplements. Notable among these are reduced Coenzyme Q10 (ubiquinol), alpha-lipoic acid (ALA), riboflavin, and L-carnitine, particularly in cases where deficiencies are identified. These supplements aim to support mitochondrial function and address metabolic imbalances associated with mitochondrial dysfunction (21).

For those with deficiencies presenting with central nervous system involvement, folinic acid is recommended. This supplementation is designed to address specific neurological aspects, offering a targeted approach for individuals facing challenges in this domain. The inclusion of folinic acid underscores the recognition of the varied clinical presentations within the spectrum of mitochondrial disorders (22).

Furthermore, individuals diagnosed with mitochondrial encephalomyopathy lactic acidosis and stroke-like episodes (MELAS) attributed to the m.3243A>G mutation are advised to consider daily oral arginine supplementation. This specific recommendation is geared toward stroke prevention, addressing a critical aspect of the clinical manifestations associated with this particular mitochondrial mutation (23).

### Idiopathic late-onset cerebellar ataxia

Idiopathic Late-Onset Cerebellar Ataxia (ILOCA) presents a diagnostic challenge, especially when symptomatic cerebellar ataxia is ruled out. In individuals below the age of 50, even in the absence of an apparent family history, it becomes essential to explore the possibility of hereditary ataxia. A systematic screening approach is recommended, beginning with recessive ataxias and subsequently examining the group of Spinocerebellar Ataxias (SCAs). In instances where all diagnostic tests yield inconclusive results, the term ILOCA is employed to describe the condition (24).

The clinical spectrum of ILOCA spans from isolated idiopathic late-onset cerebellar degeneration to more complex presentations involving the combined degeneration of the cerebellum, vestibular system, and sensory afferents. A notable example within this spectrum is the clinical entity known as Cerebellar Ataxia Neuropathy Vestibular areflexia Syndrome (CANVAS). CANVAS represents a distinctive subset characterized by the involvement of multiple neurological systems, further complicating the diagnostic landscape of late-onset cerebellar ataxias (25).

Given the heterogeneity of presentations within the idiopathic late-onset ataxia spectrum, a comprehensive diagnostic approach is crucial. This includes thorough clinical evaluations, imaging studies, and genetic testing to delineate potential hereditary factors contributing to the ataxic phenotype. The recognition of conditions like CANVAS underscores the importance of considering the broader clinical spectrum beyond isolated idiopathic late-onset cerebellar degeneration (26).

### Multiple system atrophy (MSA)

**Figure 1:**
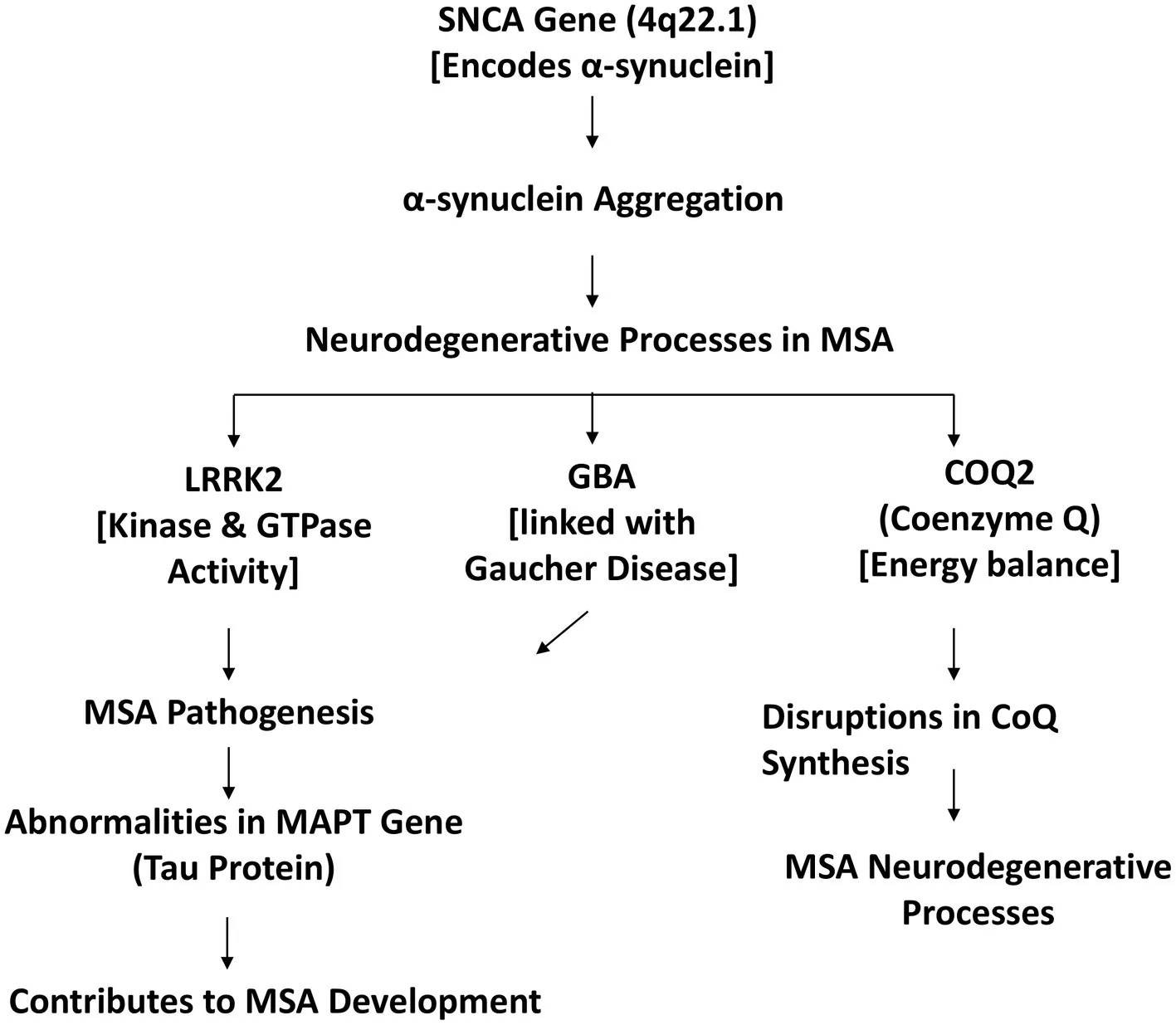
Pathways involved in the neurodegenerative processes of multiple system atrophy.

Multiple System Atrophy (MSA) stands as a challenging ɑ-synucleinopathy, a classification denoted by the presence of glial cytoplasmic inclusions (GCI) in oligodendrocytes. The distinctive pathological feature of MSA, these inclusions contribute to the progressive degeneration of neurons within the central nervous system. The intricate interplay of various genetic factors further adds complexity to the understanding of MSA’s pathogenesis (27). The molecular mechanism of MSA as depicted in Figure 1.

The SNCA gene, located at 4q22.1, takes center stage in MSA’s genetic landscape. This gene encodes the ɑ-synuclein protein, primarily found in neuronal presynaptic terminals, where it plays a crucial role in synaptic transmission (28). Abnormalities in ɑ-synuclein, leading to its aggregation, are a central theme in the neurodegenerative processes observed in MSA (29).

LRRK2, or leucine-rich repeat kinase, emerges as another pivotal player associated with MSA. This protein possesses kinase and GTPase activities, and certain populations exhibit the G2019S mutation in the LRRK2 gene, linking it notably to MSA pathogenesis (30). The implications of this mutation contribute to the intricate genetic landscape of the disease.

GBA mutations, originally associated with Gaucher Disease, unveil a connection with MSA pathogenesis. The genetic intricacies interlinking these conditions highlight the shared genetic underpinnings that can contribute to the development of neurodegenerative disorders.

Furthermore, alterations in COQ2 expression, influencing Coenzyme Q (CoQ) and ATP levels, emerge as significant contributors to MSA (31). CoQ is vital for cellular energy production, and disruptions in its synthesis may impact the energetic balance within cells, contributing to the neurodegenerative cascade observed in MSA (29).

Abnormalities in the MAPT gene, responsible for encoding the tau protein, add another layer of complexity to MSA’s genetic landscape (32). Tau protein abnormalities are commonly associated with tauopathies, and alterations in MAPT contribute to the intricate web of genetic factors influencing MSA development.

The genetic basis of Multiple System Atrophy involves a complex interplay of various genetic factors, prominently featuring the SNCA gene, LRRK2 mutations, GBA mutations, COQ2 expression alterations, and abnormalities in the MAPT gene. The understanding of these genetic intricacies contributes to ongoing research efforts aimed at unraveling the pathogenesis of MSA and developing targeted therapeutic strategies for this challenging neurodegenerative disorder (33).

### Hereditary spastic paraplegias

Hereditary Spastic Paraplegias (HSP) form a heterogeneous group of neurodegenerative disorders characterized by progressive stiffness and weakness in the leg muscles, stemming from the degeneration of axons in specific tracts of the central nervous system (34). The complexity of HSP arises from its genetic diversity, with over 80 identified genes contributing to various subtypes of the condition (35). The pathogenesis of HSP involves the disruption of essential cellular functions related to lipid metabolism, axonal transport, organelle shaping, and the endo-lysosomal system. This intricate network of processes underscores the multi-faceted nature of HSP, making it challenging to pinpoint specific therapeutic targets. Notably, HSP-related genes exert their influence on the endoplasmic reticulum, providing a potential avenue for therapeutic exploration (35, 36).

In the quest for effective interventions, compounds targeting lipid synthesis and lysosomal function have shown promise. Miglustat and resveratrol, in particular, demonstrate potential in mitigating the impact of HSP-related pathogenic mechanisms (37). However, their clinical application faces challenges, including toxicities and adverse effects that warrant careful consideration.

**Figure 2:**
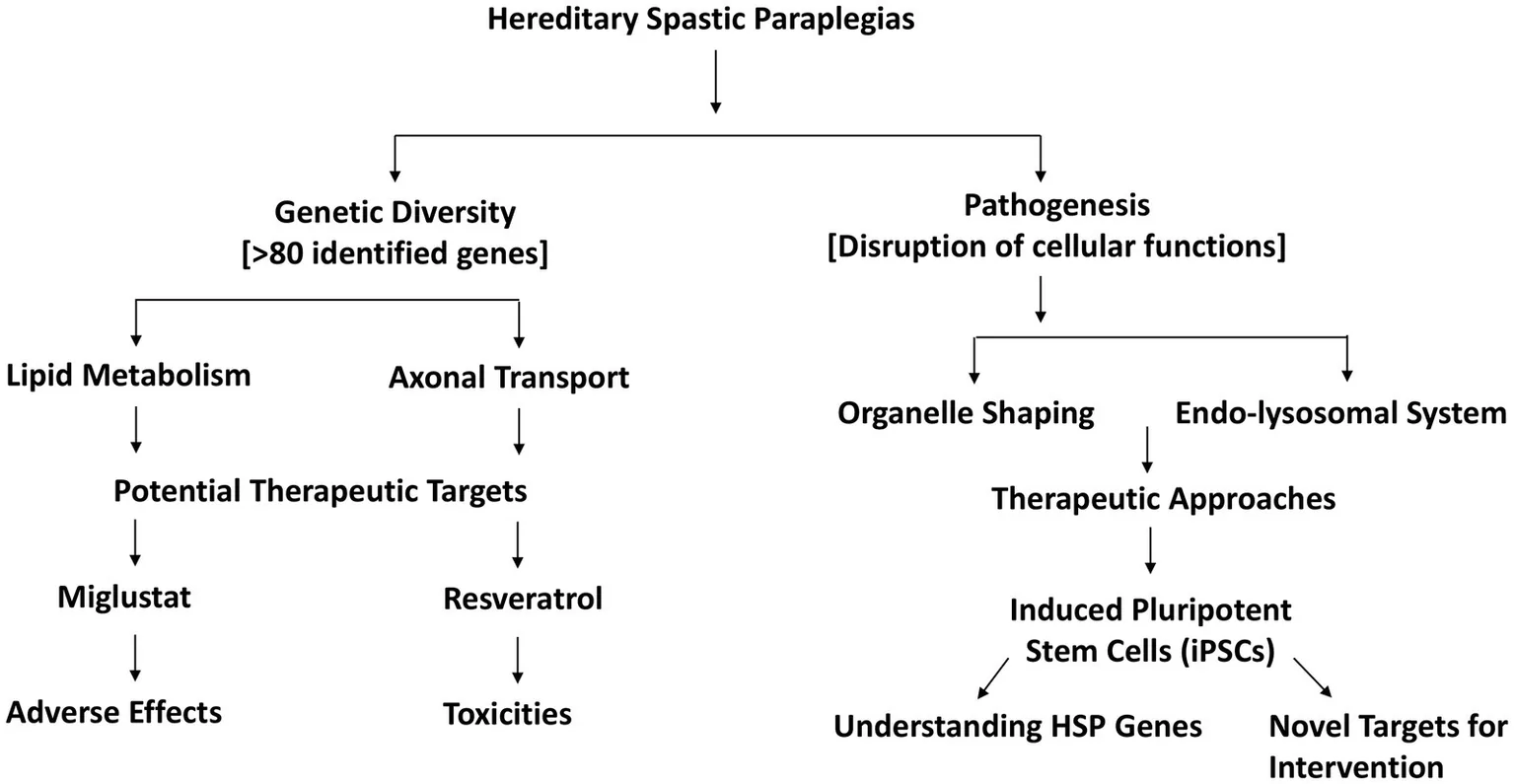
Pathophysiological mechanisms and therapeutic strategies in hereditary spastic paraplegias.

The emergence of induced pluripotent stem cells (iPSCs) has significantly contributed to our understanding of HSP genes and the evaluation of potential drug effects. iPSCs serve as a valuable tool in studying the underlying genetic factors of HSP and offer a platform for exploring and refining therapeutic approaches. This innovative approach provides researchers with a means to unravel the complexities of HSP at the cellular level, facilitating the identification of novel targets for intervention (38). The HSP-associated athophysiological mechanisms are shown in Figure 2. Hereditary Spastic Paraplegias present a complex landscape of neurodegenerative disorders driven by a multitude of genetic factors. The ongoing exploration of compounds targeting lipid synthesis and lysosomal function, along with the utilization of iPSCs for research, holds promise in advancing our understanding and potential treatment strategies for HSP. As research progresses, these efforts may pave the way for more targeted and effective therapeutic interventions for individuals affected by this challenging group of disorders (37).

### Inherited Parkinson’s disease

In the realm of Inherited Parkinson’s Disease (IPD), the understanding of specific genetic factors has shed light on the complex landscape of familial Parkinson’s disease. Notably, mutations in genes such as LRRK2 and PINK1 have emerged as key contributors, each offering unique insights into the molecular mechanisms underlying this neurodegenerative disorder. LRRK2 mutations, recognized as a common cause of autosomal dominant Parkinson’s disease, closely resemble the idiopathic form of the condition (39). The autosomal dominant inheritance pattern signifies that a single copy of the mutated gene can confer an increased risk of developing PD. LRRK2’s role in regulating various cellular processes makes it a focal point in understanding the pathogenesis of familial PD (40).

On the other hand, PINK1 mutations manifest in autosomal recessive juvenile Parkinsonism, resembling early-onset PD akin to biallelic Parkin mutations. PINK1 encodes a kinase with a crucial role in mitochondrial function, hinting at disruptions in mitochondrial dynamics as a contributing factor to PD pathology (41). The identification of such genetic mutations underscores the importance of genetic testing to unravel the mitochondrial dysfunction present in PD patients, although its utilization remains underappreciated in current clinical practice (42).

Experimental studies exploring PINK1 mutations have provided intriguing insights into potential therapeutic avenues. Increased kinase activity observed in mutant PINK1, especially with kinetin triphosphate (KTP), suggests a potential target for intervention. Additionally, experimental evidence points toward neuroprotection with Mitoquinone (MitoQ), indicating a potential avenue for mitochondrial-focused therapies. However, translating these findings into clinical success has proven challenging, with clinical trials of MitoQ showing limited efficacy (42, 43).

In the quest for gene-specific therapies, Rasagiline, a newer monoamine oxidase B (MAO-B) inhibitor, has emerged with promise for neuroprotection in PD. Its potential, especially with low-dose administration, holds implications for tailoring treatments based on the genetic profile of individuals with PD. This represents a step toward personalized medicine, aligning with the broader goal of developing targeted therapies that consider the genetic nuances of Parkinson’s disease (16).

In summary, the exploration of specific genetic mutations, such as those in LRRK2 and PINK1, provides valuable insights into the intricate landscape of Inherited Parkinson’s Disease. From autosomal dominant patterns resembling idiopathic PD to the mitochondrial involvement of PINK1, these genetic discoveries pave the way for a deeper understanding of PD pathogenesis and the development of gene-specific therapeutic strategies, ushering in a new era of precision medicine in the realm of neurodegenerative disorders (44).

**Figure 3:**
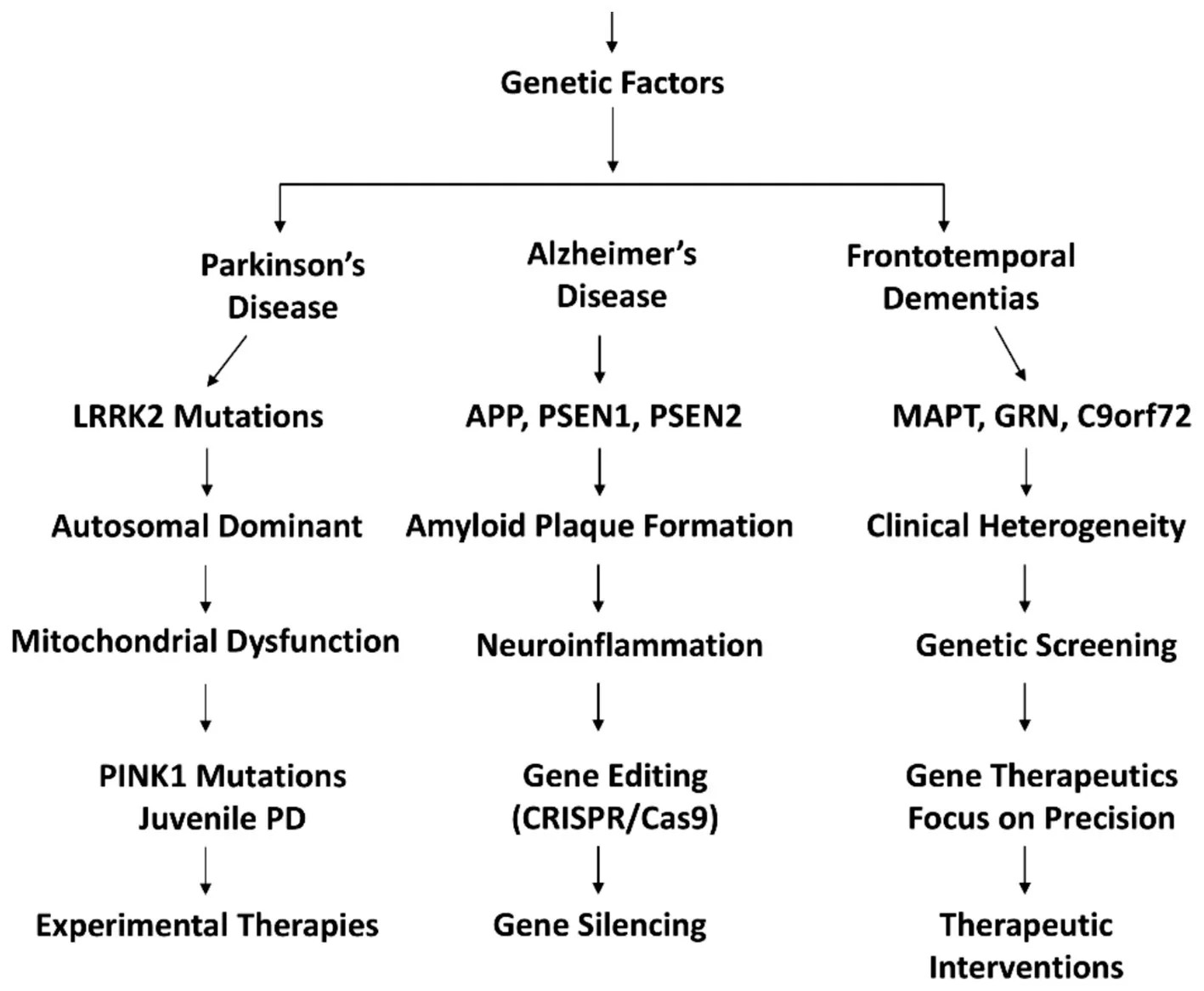
Genetic underpinnings and targeted therapeutic approaches for inherited neurodegenerative disorders: a comprehensive overview of genetic mutations and their corresponding treatment strategies.

The genetic contributions and therapeutic approaches in major inherited neurodegenerative diseases are shown in Figure 3.

### Inherited dementias

#### Alzheimer’s disease

Alzheimer’s Disease (AD) is a devastating neurological condition characterized by extensive neuronal loss, the accumulation of neurofibrillary tangles, and amyloid plaques (45).

The genetic basis of AD is multifaceted, with early-onset mutations in APP, PSEN1, and PSEN2 contributing to the acceleration of disease onset. These mutations influence the formation of amyloid plaques and the accumulation of neurofibrillary tangles, hallmark pathological features of AD (45). Late-onset variants identified through GWAS highlight the complexity of genetic susceptibility, involving genes like CR1, CD33, MS4A, CLU, ABCA7, and EPHA1 (46). Understanding the influence of these genetic factors is crucial for unraveling the intricacies of AD progression (47).

Neuroinflammation plays a pivotal role in AD pathogenesis, involving the activation of microglia and the expression of pro-inflammatory genes (48). Recent research emphasizes the potential of immune system modulation as a therapeutic approach. Studies demonstrating the anti-inflammatory properties of low-dose curcumin and the emergence of high-dose monoclonal antibodies targeting Aβ plaques showcase promising avenues for intervention. However, current therapies for AD primarily focus on symptom control, lacking proven long-term resolution or prevention of neurodegeneration (48, 49).

In the realm of gene therapeutics, the exploration of gene-editing technologies like CRISPR/Cas9 and RNA-based therapeutics holds promise. Addressing the genetic basis of AD through precision medicine approaches may offer novel treatment avenues. As research in this field advances, understanding the interplay between genetic mutations, neuroinflammation, and therapeutic interventions becomes crucial for developing effective strategies to combat Alzheimer’s Disease (50).

#### Vascular dementia

Vascular Dementia (VaD) stands as a significant neurological condition characterized by cognitive impairment arising from cerebrovascular events, predominantly strokes and ischemic damage. This exploration delves into the genetic foundations of VaD, aiming to identify key genetic mutations associated with the disease and investigate the potential of gene therapeutics for its management.

VaD, ranking second only to Alzheimer’s Disease in dementia prevalence, has a complex etiology intertwining vascular factors with genetic influences. Unraveling the genetic underpinnings becomes crucial in comprehending the intricate pathogenesis of VaD (51, 52).

Genetic studies reveal specific variants of the Apolipoprotein E (APOE) gene implicated in VaD, mirroring its role in Alzheimer’s Disease. APOE alleles influence vascular changes, contributing to cognitive decline. Furthermore, mutations in the Notch 3 gene are associated with cerebral autosomal dominant arteriopathy with subcortical infarcts and leukoencephalopathy (CADASIL), a hereditary form of VaD. Collagen gene mutations (COL4A1 and COL4A2) contribute to cerebral small vessel disease, impacting cognitive function (53).

Combining genetic screening with advanced neuroimaging techniques is instrumental in identifying mutations and assessing vascular changes. Gene expression profiling in individuals with VaD offers insights into potential therapeutic targets and changes associated with vascular dysfunction (53). Gene therapeutics for VaD focus on neuroprotection, maintaining vasculature integrity, and targeting vascular risk factors. Gene editing technologies and expression modulation hold promise in mitigating the impact of genetic mutations. Addressing challenges in translating genetic discoveries into effective therapies is critical for future developments, including personalized interventions and precision medicine approaches (53).

Recent advances in hub gene–drug interaction analysis have identified four potential drugs with promising implications for VaD treatment: maraviroc, cenicriviroc, PF-04634817, and efalizumab (54). Maraviroc is a medication primarily used to treat HIV/AIDS by inhibiting the CCR5 receptor. The identification of maraviroc in hub gene–drug interaction analysis suggests a potential role in addressing vascular dementia, possibly through modulation of inflammatory responses or neuroprotective mechanisms (55). Cenicriviroc is a drug initially developed for the treatment of HIV infection and is also being investigated for non-alcoholic steatohepatitis (NASH). Its potential in VaD treatment may indicate a connection between immune system modulation and cognitive function, warranting further exploration into its neuroprotective effects (56). PF-04634817 is a selective prostaglandin E2 receptor subtype 4 (EP4) antagonist that has been investigated for its anti-inflammatory properties. Its identification in the hub gene–drug interaction analysis implies a potential role in mitigating inflammation associated with VaD, contributing to cognitive preservation (57). Efalizumab is a monoclonal antibody initially used for the treatment of psoriasis (58).

Its recognition in hub gene–drug interaction analysis suggests a potential avenue for exploring the immunomodulatory effects of this drug in addressing neuroinflammation associated with VaD.

### Dementia with Lewy bodies

Lewy body dementia (LBD) and Dementia with Lewy Bodies (DLB) collectively represent a complex and diverse spectrum of neurodegenerative diseases characterized by cognitive decline, parkinsonism, and hallucinations (48). Advances in genome sequencing techniques offer a deeper understanding of disease-associated genetic variations, shedding light on the complex genetic landscape of these conditions. These insights into the genetic landscape of Lewy body dementia (LBD) and Dementia with Lewy Bodies (DLB) offer a comprehensive perspective on the underlying causes of these complex neurodegenerative disorders. The identification of Lewy bodies as a neuropathological hallmark and the discovery of specific genetic mutations, such as those in the alpha-synuclein (SNCA) and beta-synuclein (SNCB) genes, contribute to our understanding of the molecular mechanisms driving disease progression (59).

Lewy body dementia (LBD) and Dementia with Lewy Bodies (DLB) collectively represent a complex and diverse spectrum of neurodegenerative diseases characterized by cognitive decline, parkinsonism, and hallucinations. Advances in genome sequencing techniques offer a deeper understanding of disease-associated genetic variations, shedding light on the complex genetic landscape of these conditions (60).

These insights into the genetic landscape of Lewy body dementia (LBD) and Dementia with Lewy Bodies (DLB) offer a comprehensive perspective on the underlying causes of these complex neurodegenerative disorders. The identification of Lewy bodies as a neuropathological hallmark and the discovery of specific genetic mutations, such as those in the alpha-synuclein (SNCA) and beta-synuclein (SNCB) genes, contribute to our understanding of the molecular mechanisms driving disease progression (59, 60).

The genetic heterogeneity observed in LBD underscores the intricate nature of these conditions, reflecting a wide range of clinical manifestations and genetic variations. Genome sequencing technologies play a pivotal role in unraveling the complexities of these diseases, offering a platform to discern disease-associated genetic variations and paving the way for potential targeted therapeutic interventions. However, challenges persist in the analysis of genetic data, particularly in distinguishing causative mutations from incidental genetic variations. Additionally, the diverse clinical presentations pose obstacles to developing interventions that effectively address the range of symptoms exhibited by individuals with DLB and LBD (59, 60).

Nonetheless, the integration of genetic insights into the understanding of Lewy body dementia holds promise for future research and therapeutic developments. By uncovering the specific genetic factors contributing to the formation of Lewy bodies and the neurodegenerative processes, researchers aim to pave the way for more precise and tailored treatment strategies for individuals affected by these challenging neurocognitive disorders (61).

### Frontotemporal dementias

Frontotemporal Dementias (FTD) encompass a diverse group of neurodegenerative disorders characterized by progressive damage to the frontal and temporal lobes, presenting challenges in clinical heterogeneity (62, 63). The genetic landscape of Frontotemporal Dementias is multifaceted, with familial forms often exhibiting distinct mutations. Notably, mutations in the microtubule-associated protein tau (MAPT), progranulin (GRN), and chromosome 9 open reading frame 72 (C9orf72) genes are commonly associated with familial FTD. Advanced molecular techniques, including genetic screening and next-generation sequencing, play a pivotal role in uncovering these mutations and deciphering their role in disease pathogenesis (64).

Frontotemporal lobar degeneration diseases (FTLD) are traditionally categorized based on post-mortem neuropathological features, distinguishing between tau-positive inclusions (FTLD-tau) and ubiquitin-positive inclusions, often TDP-43-positive (FTDLD-TDP43). This categorization aids in understanding the underlying molecular processes contributing to neurodegeneration in FTD (65).

Furthermore, the association between Frontotemporal Dementia and amyotrophic lateral sclerosis (FTD-ALS) reveals shared genetic factors. The C9orf72 gene repeat expansion and TBK1 gene mutations are linked to both FTD-ALS and FTLD. In particular, C9orf72 poly(GA) aggregation is implicated in TBK1 phosphorylation and sequestration, resulting in reduced TBK1 activity and contributing to neurodegeneration. Studies in mice demonstrate that reducing TBK1 activity exacerbates poly(GA)-induced phenotypes, shedding light on potential mechanisms in FTLD and FTD-ALS (66).

The challenge in developing gene therapeutics for Frontotemporal Dementias lies in the clinical and genetic heterogeneity observed within these disorders. Tailoring interventions to address specific genetic mutations remains complex, and ethical considerations add an additional layer of complexity to the development of gene-based therapies.

In conclusion, Frontotemporal Dementias serve as a valuable model for investigating the genetic basis of neurological conditions. Identifying genetic mutations associated with FTD not only enhances diagnostic precision but also provides critical insights into potential gene therapeutics, shedding light on the underlying causes of these complex and challenging neurodegenerative disorders (67).

### Inherited prion diseases

Prion diseases, known for their fatal and contagious nature, have gained notoriety within the realm of neurological conditions. The most prevalent human prion disorder is sporadic Creutzfeldt-Jakob disease (sCJD), exhibiting an incidence of 1 in a million. Additionally, Gerstmann-Straussler disease (GSD), an autosomal inherited prion disorder, is linked to mutations in the prion protein gene (PRNP). Specifically, the D178N mutation, often paired with the M129V mutation in PRNP, is associated with CJD (68, 69).

Inherited Prion Diseases (IPDs), such as familial Creutzfeldt-Jakob Disease (fCJD), Gerstmann-Sträussler-Scheinker syndrome (GSS), and Fatal Familial Insomnia (FFI), constitute a subset of prion disorders caused by mutations in PRNP. These mutations lead to the abnormal accumulation of misfolded prion proteins, resulting in severe neuronal damage and progressive neurodegeneration (69).

Genetic analysis plays a pivotal role in identifying specific mutations in PRNP associated with Inherited Prion Diseases. Mutations, including substitutions at codon 200 (E200K) and codon 129 (M129V), contribute to the heterogeneity within this group of disorders. The correlation between distinct mutations and clinical phenotypes enhances our understanding of the genetic basis of Inherited Prion Diseases (70). Given the hereditary nature of these prion disorders, exploring gene therapeutics becomes a potential avenue for intervention. However, the unique challenges posed by prion diseases, where misfolded proteins induce further misfolding, complicate the development of gene-based treatments. Strategies involving gene editing techniques like CRISPR/Cas9 aim to correct pathogenic mutations or enhance cellular clearance mechanisms, yet these approaches are in early stages of development and face obstacles in terms of precision and safety (70, 71).

Prion diseases, with their distinct and ominous reputation, emphasize the importance of unraveling the genetic basis of neurological conditions. The prevalence of sCJD and the association of specific mutations, such as D178N and M129V in PRNP, underscore the need for continued research into these rare and devastating neurodegenerative disorders. Identifying genetic mutations associated with Inherited Prion Diseases not only enhances diagnostic precision but also lays the foundation for potential gene therapeutics, offering a glimpse of hope for addressing the underlying causes of these challenging conditions (71).

The development of RNA-based therapies, including RNA interference (RNAi) and antisense oligonucleotides (ASOs), offers a novel approach to treating genetic neurodegenerative disorders. These therapies can target the underlying genetic mutations responsible for abnormal protein production, offering disease-modifying potential as shown in Table 3.

**Table 3:** RNA-based therapies for neurological disorders.

Therapy type	Mechanism of action	Target disorder	Clinical stage
RNA Interference (RNAi)	Silencing of specific mRNA sequences	Huntington’s Disease	Phase III Trials
Antisense Oligonucleotides (ASOs)	Prevents translation of mutant proteins	Amyotrophic Lateral Sclerosis (ALS)	FDA Approved (Spinraza for SMA)
mRNA Vaccines	Encodes therapeutic proteins	Alzheimer’s Disease	Preclinical Research
Antisense Oligonucleotides (ASOs)	Modulates gene expression to reduce protein levels	Hereditary Transthyretin-Mediated Amyloidosis	Phase III Trials
Antisense Oligonucleotides (ASOs)	Inhibits production of toxic proteins	Frontotemporal Dementia	Phase II Trials
Antisense Oligonucleotides (ASOs)	Targets specific RNA sequences to reduce protein expression	Spinal Muscular Atrophy (SMA)	FDA Approved (Spinraza for SMA)
Antisense Oligonucleotides (ASOs)	Modulates gene expression to reduce protein levels	Duchenne Muscular Dystrophy	Phase II Trials
Antisense Oligonucleotides (ASOs)	Inhibits production of toxic proteins	Amyotrophic Lateral Sclerosis (ALS)	Phase I Trials
Antisense Oligonucleotides (ASOs)	Targets specific RNA sequences to reduce protein expression	Huntington’s Disease	Phase I Trials
Antisense Oligonucleotides (ASOs)	Modulates gene expression to reduce protein levels	Parkinson’s Disease	Phase I Trials
Antisense Oligonucleotides (ASOs)	Inhibits production of toxic proteins	Alzheimer’s Disease	Phase I Trials
Antisense Oligonucleotides (ASOs)	Targets specific RNA sequences to reduce protein expression	Amyotrophic Lateral Sclerosis (ALS)	Phase I Trials
Antisense Oligonucleotides (ASOs)	Modulates gene expression to reduce protein levels	Spinal Muscular Atrophy (SMA)	Phase I Trials
Antisense Oligonucleotides (ASOs)	Inhibits production of toxic proteins	Duchenne Muscular Dystrophy	Phase I Trials
Antisense Oligonucleotides (ASOs)	Targets specific RNA sequences to reduce protein expression	Frontotemporal Dementia	Phase I Trials
Antisense Oligonucleotides (ASOs)	Modulates gene expression to reduce protein levels	Hereditary Transthyretin-Mediated Amyloidosis	Phase I Trials
Antisense Oligonucleotides (ASOs)	Inhibits production of toxic proteins	Huntington’s Disease	Phase I Trials
Antisense Oligonucleotides (ASOs)	Targets specific RNA sequences to reduce protein expression	Parkinson’s Disease	Phase I Trials
Antisense Oligonucleotides (ASOs)	Modulates gene expression to reduce protein levels	Alzheimer’s Disease	Phase I Trials
Antisense Oligonucleotides (ASOs)	Inhibits production of toxic proteins	Amyotrophic Lateral Sclerosis (ALS)	Phase I Trials
Antisense Oligonucleotides (ASOs)	Targets specific RNA sequences to reduce protein expression	Spinal Muscular Atrophy (SMA)	Phase I Trials
Antisense Oligonucleotides (ASOs)	Modulates gene expression to reduce protein levels	Duchenne Muscular Dystrophy	Phase I Trials
Antisense Oligonucleotides (ASOs)	Inhibits production of toxic proteins	Frontotemporal Dementia	Phase I Trials
Antisense Oligonucleotides (ASOs)	Targets specific RNA sequences to reduce protein expression	Hereditary Transthyretin-Mediated Amyloidosis	Phase I Trials

### Huntington’s disease

Huntington’s Disease (HD) is a genetic disorder characterized by movement and cognitive impairments, including chorea and coordination loss, stemming from mutations in the HTT gene encoding huntingtin (72). The distinct clinical features of HD arise from the abnormal expansion of CAG repeats in the HTT gene, leading to the production of a mutated huntingtin protein. This polyglutamine expansion triggers a cascade of events, disrupting cellular functions and ultimately causing the characteristic symptoms of HD (73, 74).

Currently, treatment options for HD are limited. Tetrabenazine, an approved drug, helps manage symptoms such as chorea by reducing dopamine levels in the brain. However, it does not alter the course of the disease. The search for effective therapeutics has led to exploring gene silencing strategies aimed at reducing the expression of mutant huntingtin. This approach holds promise in slowing disease progression or preventing its onset, making it a focal point in ongoing research and clinical trials (74, 75).

Minocycline, an antibiotic known for its caspase inhibitory properties, has demonstrated potential in extending life expectancy in mouse models of HD. While results vary, this finding suggests that targeting specific cellular processes associated with HD pathology may hold therapeutic value. Further research is needed to elucidate the precise mechanisms and assess the translation of these findings to human subjects (74, 76). The genetic basis of HD, characterized by mutations in the HTT gene, provides a unique avenue for exploring therapeutic interventions (69–72). The current landscape includes approved drugs for symptom management and promising avenues, such as gene silencing strategies and potential disease-modifying agents like minocycline, offering hope for improved treatment outcomes in the future (79, 80).

### Impact of next generation sequencing on the understanding of neurological disorders

Next Generation Sequencing (NGS) technologies allow for the comprehensive identification of genetic mutations linked to neurological diseases, as ashown in Table 4. By sequencing entire genomes, NGS has enabled the discovery of novel genes associated with diseases like Parkinson’s, Alzheimer’s, and Epilepsy. The integration of RNA-based therapeutics and NGS technologies is revolutionizing the landscape of neurological disorder treatment. While RNA-based therapies offer targeted interventions at the genetic level, NGS allows for the precise identification of disease-causing mutations. These combined advances hold the promise of earlier diagnosis, better-targeted therapies, and ultimately, improved outcomes for patients with debilitating neurological conditions. It is interesting how innovations such as single-cell RNA sequencing and long-read sequencing have enhanced our ability to identify previously elusive genetic mutations (15, 16, 77). These advancements have significantly improved our understanding of the complex genetic architectures underlying hereditary ataxias and other neurodegenerative diseases. Recent findings indicate that long-read sequencing is particularly effective in resolving repeat expansions, a common feature in diseases like Spinocerebellar ataxias (SCAs), further demonstrating the transformative role of NGS in diagnostics and treatment strategies (16, 17, 78).

**Table 4:** Impact of next generation sequencing (NGS) on neurological disorders and the genes involved.

Disorder	Genes identified	Impact of NGS
Parkinson’s Disease	LRRK2, PARK7, PINK1, SNCA	NGS has facilitated the identification of novel mutations, providing insights into genetic predisposition and offering potential for early genetic testing and personalized therapies.
Alzheimer’s Disease	APP, PSEN1, PSEN2, CR1, APOE, CLU	NGS has enabled the identification of rare genetic variants and biomarkers for early diagnosis, helping to refine diagnostic criteria and create gene-targeted therapeutic approaches.
Epilepsy	SCN1A, KCNQ2, PCDH19, GABRG2	NGS has improved the understanding of genetic mutations that underlie epileptic syndromes, aiding in personalized treatment plans and contributing to more accurate diagnosis.
Spinocerebellar Ataxias (SCAs)	ATXN1, ATXN2, ATXN3	Long-read sequencing helps resolve repeat expansions that are difficult to detect with traditional methods, enabling the identification of mutations causing SCA and paving the way for targeted therapies.
Huntington’s Disease	HTT (Huntingtin gene)	NGS has accelerated the identification of expanded CAG repeats and supported genetic counseling for patients and their families. Moreover, it aids in assessing therapeutic response to gene silencing.
Amyotrophic Lateral Sclerosis (ALS)	SOD1, C9orf72, FUS, TDP-43	NGS has allowed the detection of previously undiagnosed genetic mutations in ALS patients, providing new insights into disease mechanisms and advancing precision medicine for ALS.
Hereditary Ataxias	FXN (Friedreich’s Ataxia), POLG, MT-TL1	NGS technologies have revealed new gene mutations, contributing to more precise diagnostic techniques and potentially opening up new therapeutic options based on gene editing and mutation-targeted approaches.
Multiple Sclerosis	IL7R, TNF, CD4, CD8, APOE	NGS enables the exploration of immune-related genes that contribute to multiple sclerosis, leading to the identification of new biomarkers for risk prediction and potential immunomodulatory therapies.
Schizophrenia	DISC1, COMT, NRG1, BDNF, AKT1	NGS has revealed the involvement of rare genetic variants, contributing to a better understanding of the neurodevelopmental and synaptic plasticity disruptions in schizophrenia.
Fragile X Syndrome	FMR1	NGS has provided insights into the identification of CGG repeat expansions in the FMR1 gene, contributing to early diagnosis and the exploration of potential gene silencing therapies.
Prion Diseases	PRNP	NGS technologies have improved our ability to detect prion protein mutations, contributing to a deeper understanding of prion pathogenesis and potential therapeutic avenues to target protein aggregation.
Frontotemporal Dementia (FTD)	MAPT, GRN, C9orf72	NGS helps identify the underlying genetic causes of FTD, especially rare mutations such as in MAPT and GRN genes, which opens new avenues for targeted genetic therapies and early diagnosis.
Multiple System Atrophy (MSA)	SNCA, LRRK2, GBA, COQ2, MAPT	NGS has uncovered genetic mutations and provided a more thorough understanding of the genetic architecture behind MSA, offering insights into possible therapeutic targets like α-synuclein aggregation inhibition.

## Discussion

The landscape of neurodegenerative disorders has seen a paradigm shift with the advent of modern therapeutic techniques. Given the intricate genetic foundations of many neurological conditions, innovative treatment approaches are now more crucial than ever. The current study integrates the latest genetic research, therapeutic advancements, and emerging technologies to create a framework for addressing the global burden of neurodegenerative diseases (10–15). By addressing the pressing issue of the global burden of neurological conditions, particularly their impact on cognitive and motor functions, the study lays a foundation for innovative approaches and prospects in the field. The global burden of neurological disorders underscores the urgency to address these conditions, particularly in the context of their increasing prevalence in low-income and middle-income countries. This contextualization provides a comprehensive backdrop for the subsequent exploration of genetic and therapeutic aspects.

The exploration of rare hereditary neurodegenerative disorders underscores the profound genetic complexity of these conditions. Advances in NGS have uncovered numerous gene mutations responsible for cognitive and motor impairments. By classifying genes into causative and associated categories, we gain a better understanding of how mutations not only contribute to disease pathology but also to species diversity. These genetic insights have paved the way for precision medicine, where individualized treatment plans can be developed based on a patient’s specific genetic profile. This approach holds promise in tailoring interventions that maximize therapeutic efficacy while minimizing side effects (26–37).

The exploration of RNA-based therapeutics, particularly in the context of neurological disorders associated with RNA metabolism, opens promising avenues for treating complex conditions like brain tumors, stroke, and neurodegenerative diseases. The classification of genes into causative and associated, with mutations considered as contributors to species diversity, provides a foundational understanding of the genetic underpinnings of these disorders. Through mechanisms such as RNA interference and antisense oligonucleotides, RNA-based therapies can effectively modulate gene expression, offering a targeted approach to disease management. Moreover, these therapies can be engineered to have temporal and spatial precision, thereby enhancing their efficacy in reaching specific neural tissues (48, 58).

Gene therapy has rapidly evolved into a cornerstone of modern neurology research. The delivery of genetic material directly into the brain via neurosurgical techniques has become a key strategy in treating diseases such as spinal muscular atrophy and Huntington’s disease. One of the most innovative aspects of gene therapy is the ability to deliver therapeutic genes with temporal and spatial precision. Advances in neurosurgical tools and imaging techniques, such as intraoperative MRI, have enabled more accurate delivery of therapeutic agents. These interventions target specific regions of the brain, reducing off-target effects and improving patient outcomes (35, 37–45).

Recent advancements in neuroimaging technologies, such as functional MRI and positron emission tomography, have revolutionized the diagnosis and treatment of neurological disorders. By identifying disease-specific biomarkers, these imaging techniques provide valuable insights into the progression of neurodegenerative diseases. The integration of genetic data with neuroimaging allows for the development of personalized treatment plans. For instance, in conditions such as multiple sclerosis, neuroimaging biomarkers can help predict treatment response, facilitating more precise therapeutic interventions (54).

Neurological disorders impose a significant global burden, particularly in low- and middle-income countries where access to advanced healthcare is limited. The rising prevalence of these disorders in these regions calls for a global strategy that combines cutting-edge research with accessible healthcare solutions. By fostering international collaboration, sharing genetic data, and implementing cost-effective therapies, we can address the global impact of neurodegenerative diseases. Programs focused on public health education and early diagnosis in underserved areas will be essential in reducing the burden of neurological conditions (25–32).

The multifaceted nature of neurological disorders necessitates a multidisciplinary approach to treatment. By combining genetic studies, emerging therapeutic strategies, and neuroimaging techniques, we can create a more holistic framework for addressing these complex conditions (81). Collaboration between geneticists, neurologists, and neurosurgeons will be crucial in advancing both our understanding and treatment of neurodegenerative diseases. Moving forward innovative approaches such as, expanding the use of artificial intelligence (AI) in analyzing genetic data, developing more efficient gene-editing technologies like CRISPR-Cas9, and enhancing RNA-based therapeutic delivery systems should be promoted. Furthermore, emphasizing the potential of gene therapy, highlighting factors such as temporal and spatial precision in targeted delivery through neurosurgery. The aforementioned multifaceted approaches, combining genetic studies, neuroimaging techniques, and emerging therapeutic strategies, sets the stage for continued advancements in understanding and treating these complex neurological disorders. By embracing these innovative strategies, we can significantly advance the treatment of rare hereditary neurodegenerative conditions and improve patient outcomes worldwide.
